# Assessing the Predictive Validity of Simple Dementia Risk Models in Harmonized Stroke Cohorts

**DOI:** 10.1161/STROKEAHA.120.027473

**Published:** 2020-06-17

**Authors:** Eugene Y.H. Tang, Christopher I. Price, Louise Robinson, Catherine Exley, David W. Desmond, Sebastian Köhler, Julie Staals, Bonnie Yin Ka Lam, Adrian Wong, Vincent Mok, Regis Bordet, Anne-Marie Bordet, Thibaut Dondaine, Jessica W. Lo, Perminder S. Sachdev, Blossom C.M. Stephan

**Affiliations:** 1 Population Health Sciences Institute, Newcastle University, Campus for Ageing and Vitality, Newcastle Upon Tyne, United Kingdom (E.Y.H.T., C.I.P., L.R., C.E.).; 2 School for Mental Health and Neuroscience, Maastricht University, the Netherlands (S.K.).; 3 Department of Neurology, Cardiovascular Research Institute Maastricht, Maastricht University Medical Center, the Netherlands (J.S.).; 4 Division of Neurology, Department of Medicine and Therapeutics, Faculty of Medicine, Gerald Choa Neuroscience Centre, Therese Pei Fong Chow Research Centre for Prevention of Dementia, Lui Che Woo Institute of Innovative Medicine, The Chinese University of Hong Kong SAR (B.Y.K.L., A.W., V.M.).; 5 University Lille, Inserm, CHU Lille, U1171–Degenerative and Vascular Cognitive Disorders, France (R.B., A.-M.B., T.D.).; 6 Centre for Healthy Brain Ageing, School of Psychiatry, University of New South Wales, Sydney, Australia (J.W.L., P.S.S.).; 7 Neuropsychiatric Institute, Prince of Wales Hospital, Sydney (P.S.S.).; 8 Institute of Mental Health, Division of Psychiatry and Applied Psychology, School of Medicine, Nottingham University, UK (B.C.M.S.).

**Keywords:** aging, dementia, follow-up studies, risk prediction, risk factors

## Abstract

Stroke is associated with an increased risk of dementia. To assist in the early identification of individuals at high risk of future dementia, numerous prediction models have been developed for use in the general population. However, it is not known whether such models also provide accurate predictions among stroke patients. Therefore, the aim of this study was to determine whether existing dementia risk prediction models that were developed for use in the general population can also be applied to individuals with a history of stroke to predict poststroke dementia with equivalent predictive validity.

**Methods:**

Data were harmonized from 4 stroke studies (follow-up range, ≈12–18 months poststroke) from Hong Kong, the United States, the Netherlands, and France. Regression analysis was used to test 3 risk prediction models: the Cardiovascular Risk Factors, Aging and Dementia score, the Australian National University Alzheimer Disease Risk Index, and the Brief Dementia Screening Indicator. Model performance or discrimination accuracy was assessed using the C statistic or area under the curve. Calibration was tested using the Grønnesby and Borgan and the goodness-of-fit tests.

**Results:**

The predictive accuracy of the models varied but was generally low compared with the original development cohorts, with the Australian National University Alzheimer Disease Risk Index (C-statistic, 0.66) and the Brief Dementia Screening Indicator (C-statistic, 0.61) both performing better than the Cardiovascular Risk Factors, Aging and Dementia score (area under the curve, 0.53).

**Conclusions:**

Dementia risk prediction models developed for the general population do not perform well in individuals with stroke. Their poor performance could have been due to the need for additional or different predictors related to stroke and vascular risk factors or methodological differences across studies (eg, length of follow-up, age distribution). Future work is needed to develop simple and cost-effective risk prediction models specific to poststroke dementia.

## Abstract 2

Supplemental Digital Content is available in the text.

Cognitive impairment is common after stroke,^1^ but stroke is also a strong independent risk factor for all-cause dementia.^2^ Approximately 10% of patients develop dementia soon after a first stroke, and more than a third have dementia after a recurrent stroke.^3^ The incidence of dementia after stroke is ≈50× higher in the year after a major stroke compared with the general population.^4^ Increasing numbers of patients are surviving a stroke, and this will lead to increasing numbers of patients later developing dementia. Reliable identification of those at high risk could lead to improved risk factor management, better anticipation of care needs, and an increased opportunity to benefit from interventions to reduce dementia risk,^5^ including lifestyle-based interventions recently emphasized by the World Health Organization to delay or prevent cognitive decline and dementia.^6^

Poststroke dementia can be defined as any form of dementia that develops following a clinical cerebrovascular event.^7^ Systematic reviews have identified over 20 different dementia risk prediction models, and they have been shown to differ in their predictive accuracy.^8–10^ However, few models have been developed in a clinical setting and even fewer have been externally validated, and those that have been externally validated have had mixed results.^9,11–14^ This raises the question of the applicability of such models outside the settings in which they were developed. Within the context of stroke, only 1 model has been developed to predict poststroke dementia^15^ and 2 models have been developed to predict poststroke cognitive impairment.^16,17^ While their predictive accuracy was found to be acceptable, the utility of such models is questionable because they all include neuroimaging variables that are costly to obtain and not easily accessible, particularly in research settings or primary care where the majority of stroke patients are followed. In addition, a previous population-based cohort study found neuroimaging variables did not significantly improve dementia risk prediction accuracy when added to a simple model incorporating sociodemographic, cognitive, health, lifestyle, functional, and genetic predictors.^18^ Within the context of stroke, it is unclear whether simple prediction models excluding neuroimaging variables may be sufficient. Further, it is unknown whether dementia risk prediction models developed for the whole population will also offer accurate predictions in individuals with stroke. Thus, the aim of this study was to assess whether simple models developed to predict incident dementia in the general population also offer accurate predictions in stroke patients, which would thus permit the use of a single model to predict dementia regardless of disease subgroup.

## Methods

### Data Availability

The data that support the findings of this study are available from the corresponding author upon reasonable request.

### Dementia Risk Models Selected for Testing

Dementia risk prediction models were selected from previous systematic reviews.^8–10^ Four criteria were used to select models: (1) published predictive accuracy as measured using the C statistic or area under the curve was acceptable at ≥0.70; (2) the models incorporated variables that could be simply and easily collected in primary care and research settings (ie, models that included neuroimaging variables were not considered); (3) similar or near similar predictor variables were available in our dataset; and (4) the models included both nonmodifiable and modifiable risk factors to allow for possible future work focused on the development of risk reduction strategies. Based on these criteria, 3 models were selected: (1) the Australian National University Alzheimer Disease Risk Index (ANU-ADRI) common variables model,^14,19^ (2) the Cardiovascular Risk Factors, Aging and Dementia (CAIDE) score,^20^ and (3) the Brief Dementia Screening Indicator (BDSI).^21^ Full details of each model are included in Table 1. Whenever possible, we used the same variables that were included in the original models. When the stroke cohorts in our study did not have the same variable, substitute variables were used. Full details of how the variables in the models were mapped in each stroke cohort and any variable substitutions that were made are provided in Table I in the Data Supplement.

**Table 1.**
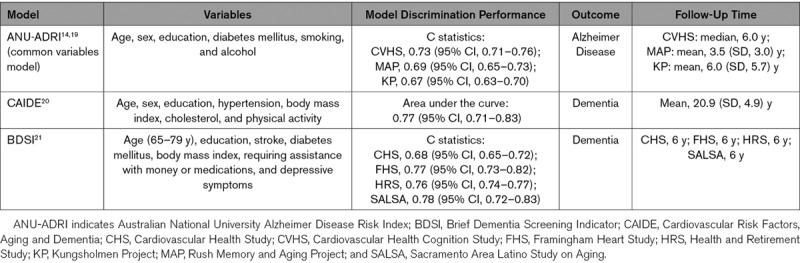
Description of Each Model Being Externally Validated

### Stroke Patient Sources

Individual participant data were obtained via the STROKOG (Stroke and Cognition Consortium)—an international consortium of longitudinal poststroke studies from around the world (Table II in the Data Supplement).^22^ To maximize the sample size and the number of incident dementia cases to adequately test the models, we harmonized data from multiple cohorts in the STROKOG study. Four datasets were included in this analysis, but they did not all have a full set of the risk variables that we needed to test each model. Therefore, model testing is based on harmonization of different combinations of the 4 selected datasets as outlined below.

### CU-STRIDE (Hong Kong)

STRIDE (Chinese University of Hong Kong–Stroke Registry Investigating Cognitive Decline) is a large cohort study (n=1007; age range, 20–98)^23^ that enrolled patients consecutively admitted to an acute stroke unit of a university-affiliated hospital due to either a stroke or transient ischemic attack (TIA), excluding patients with prestroke dementia. To exclude patients with dementia before the index event, neurologists inquired about the patient’s cognitive function before stroke.^23^ Baseline demographic and vascular risk factors were collected, and neuropsychological assessments were performed 3 to 6 months after the index event. Participants were followed for 5 years. Psychologists administered the Clinical Dementia Rating scale, with patients suspected of having dementia being assigned a Clinical Dementia Rating of one point or more, and the diagnosis of dementia was confirmed according to the Diagnostic and Statistical Manual of Mental Disorders, Fourth Edition, criteria.

### EpiUSA (the United States)

Consecutive ischemic stroke patients ≥60 years of age (n=585) and admitted to the Columbia-Presbyterian Medical Center were recruited and followed annually for up to 10 years.^24^ Initial assessments (excluding dementia diagnosis) were conducted 7 to 10 days after stroke. Neurologists specializing in stroke administered a structured neurological examination and documented any history of cerebrovascular disease and vascular risk factors. Three months and then annually after stroke, neuropsychological and functional assessments were performed, and dementia was diagnosed based on the Diagnostic and Statistical Manual of Mental Disorders, Third Edition, Revised criteria. Following the exclusion of those who were not eligible for this study (eg, those who had dementia at baseline), 262 patients were included in the present analyses.

### CASPER (the Netherlands)

The CASPER study (Cognition and Affect After Stroke: Prospective Evaluation of Risks) is a prospective cohort study (n=246; age range, 42–91) of hospital-based patients consecutively admitted with a stroke.^25^ Baseline assessments were performed 10 to 12 weeks poststroke, with follow-up assessments at 6 and 12 months. Based on the Diagnostic Criteria for Vascular Cognitive Disorders: A VASCOG Statement,^26^ patients with a vascular cognitive disorder (VCD) were identified, with major VCD being synonymous with dementia. It should be noted that vascular disease is considered the dominant, if not the exclusive, pathology in VCD.

### STROKDEM (France)

STROKDEM (Study of Factors Influencing Post-Stroke Dementia) is a 5-year observational multicenter hospital-based prospective follow-up study of a stroke population (n=200; age range, 25–87) without dementia (https://www.clinicaltrials.gov; unique identifier: NCT01330160). Baseline and follow-up (6, 12, 36, and 60 months) evaluations included detailed cognitive and functional assessment. The Clinical Dementia Rating was used as a measure of cognitive impairment. Diagnosis of dementia was based on neurological and general clinical examinations and activities of daily living.

### Incident Dementia

Dementia diagnoses, based on each study’s definition of dementia, were recorded ≈12 to 18 months poststroke for each cohort. Although some studies had longer follow-up, a common follow-up time was used across the cohorts.

### Inclusion Criteria

Since we wanted to assess the predictors of incident dementia after stroke, we excluded patients with TIA from the analyses. Individuals with dementia at baseline were also excluded when that information was available. We then performed our analyses in 2 rounds. The first round included all patients regardless of a history of stroke or subsequent recurrent stroke or TIA as this would be a better reflection of a stroke sample in clinical practice. In the second round, to perform a sensitivity analysis, those with a history of stroke, TIA, or intracranial hemorrhage were excluded. This would enable us to determine whether the model performance would improve in a distinct subset of stroke patients. Those who had a recurrent stroke between the baseline assessment and a follow-up assessment were also excluded from this second round of analyses when that information was available. A sensitivity analysis was done to ascertain whether there was a difference between those with a single versus recurrent episodes of stroke.

### Statistical Analyses

All analyses were performed using Stata, version 15/16. Risk scores were calculated for each patient based on the 3 models and then assessed in the combined cohort as predictors of incident dementia over 12 to 18 months of follow-up. The main evaluative measures of model performance and comparison were discrimination and calibration. Discrimination refers to the ability of the model to accurately identify in 2 patients the one with and the one without the desired outcome.^27^ Calibration refers to whether the observed outcomes and predictions agree.^27^ We used the analytic methods that were chosen in the original publications to assess model performance. For example, logistic regression was used to assess the CAIDE score and Cox regression was used to assess the BDSI and ANU-ADRI scores. Discrimination was measured using the C statistic (Cox regression) or area under the curve (logistic regression). Calibration or model goodness of fit was tested using the χ^2^ statistic for the logistic regression model and the Grønnesby and Borgan test for the Cox models. We did a complete case analysis for each risk score, that is, we only included patients if they contained all the variables of the model. Sensitivity analysis found some differences in the demographic characteristics (ie, age, sex, and educational status) between those individuals included and those individuals excluded from the analysis (Table III in the Data Supplement).

## Ethics

This study was approved by Newcastle University’s Faculty of Medical Sciences Ethical Committee. The work of STROKOG has ethical approval from the University of New South Wales’ Human Research Ethics Executive. The CU-STRIDE study was approved by the Chinese University of Hong Kong–North Territories East Cluster Clinical Research Ethics Committee. EpiUSA (Epidemiologic Study of the Risk of Dementia After Stroke) was approved by the Institutional Review Board of the Columbia-Presbyterian Medical Center. The CASPER study was approved by the Medical Ethics Committee of Maastricht University Medical Center. The data provided by each study team were anonymized, and, therefore, consent was not needed.

## Results

Demographics for the individual and harmonized cohorts are shown in Table 2. The total harmonized sample included 1285 stroke patients with a mean age of 68.0 (SD, 10.8) years; 42.7% women. The mean follow-up was 364.3 days (SD, 52.9).

**Table 2.**
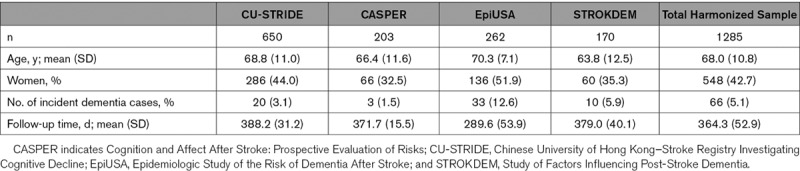
Overview of Individual Stroke Cohorts and the Total Harmonized Cohort

### Missing Data

The proportions of missing data among the predictive variables included in each model were small to moderate (range, 0%–20.7%). See Tables IV through VI in the Data Supplement for full details of the missing data. For the BDSI model, 282 of the 406 eligible participants had complete data to evaluate the model. For the ANU-ADRI model, 1065 of the 1115 eligible participants had complete data to evaluate the model. For the CAIDE model, 873 of the 1082 eligible participants had complete data to evaluate the model.

### Brief Dementia Screening Indicator

Two cohorts (EpiUSA and CASPER) were used to create the harmonized dataset for testing the BDSI model. An overview of the baseline variables is shown in Table IV in the Data Supplement. The sample included 282 patients (mean age, 65.9 years), of whom 16 developed dementia (mean follow-up, 336.0; SD, 51.7 days). The discriminative performance (C statistic) of the BDSI as mapped in the original development study and the harmonized dataset is shown in the Figure. Compared with the development cohort, the discrimination of the BDSI was low, with a wide CI (C statistic, 0.61 [95% CI, 0.42–0.79]). The model was well calibrated (χ^2^=1.26; *P*=0.26).

**Figure.**
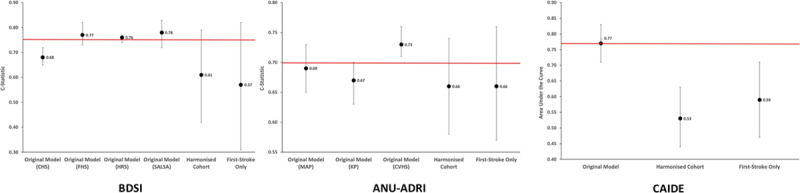
**Performance of each model in the harmonized dataset**. Red line is the mean of the original development cohort area under the curve or C statistic. ANU-ADRI indicates Australian National University Alzheimer Disease Risk Index; BDSI, Brief Dementia Screening Indicator; CAIDE, Cardiovascular Risk Factors, Aging and Dementia score; CHS, Cardiovascular Health Study; CVHS, Cardiovascular Health Cognition Study; FHS, Framingham Heart Study; HRS, Health and Retirement Study; KP, Kungsholmen Project; MAP, Rush Memory and Aging Project; and SALSA, Sacramento Area Latino Study on Aging.

### ANU-ADRI (Common Variables Model)

Three cohorts (EpiUSA, CASPER, and CU-STRIDE) were used to create the harmonized dataset for testing the ANU-ADRI model. An overview of the baseline variables is shown in Table V in the Data Supplement. The sample included 1065 patients (mean age, 68.6 years), of whom 53 developed incident dementia (mean follow-up, 362.4 days; SD, 54.2). The discriminative performance of the ANU-ADRI as mapped in the original development study and the harmonized dataset is shown in the Figure. Predictive accuracy was low (C statistic, 0.66 [95% CI, 0.58–0.74]). Similar to the BDSI, the CIs were wide. The model was well calibrated (χ^2^, 1.34; *P*=0.25).

### CAIDE Score

Three cohorts (EpiUSA, CU-STRIDE, STROKDEM) were used to create the harmonized dataset for testing the CAIDE score. An overview of the baseline variables is shown in Table VI in the Data Supplement. The sample included 873 patients (mean age, 67.6 years), of whom 43 developed incident dementia (mean follow-up, 370.4 days; SD, 51.0). The discriminative performance of the CAIDE score as mapped in the original development study and the harmonized dataset is shown in the Figure. The discriminative ability of the CAIDE score was poor for predicting incident dementia (area under the curve, 0.53 [95% CI, 0.44–0.63]). The model was well calibrated (χ^2^, 13.7; *P*=0.39).

### Sensitivity Analysis

We also performed a sensitivity analysis based on patients who had had only a single stroke (Tables IV through VI in the Data Supplement; Figure). The discriminative accuracy of these models was not significantly differently compared with the total stroke cohorts, and all models were well calibrated (all *P*>0.05).

## Discussion

In this study, we used harmonized data from STROKOG to test whether 3 dementia risk prediction models developed for use in the general population would accurately predict the incidence of dementia after stroke. This would have important implications because the identification of those at the greatest risk of dementia could allow interventions to be developed to delay its onset or even reduce its incidence. We tested existing risk models in the hope that a single model could be used regardless of comorbidities such as stroke, but predictive performance at ≈12 to 18 months poststroke was low (ie, <0.70) and poorer than the performance of the original development studies.

Our study replicates previous external validation work showing poor prediction of dementia using the CAIDE score,^12,14^ extending this work for the first time to individuals with stroke. This is likely to be due, in part, to methodologic differences between studies and is unsurprising considering the change in population and study duration. In the development study, the mean age of participants was 50.4 years (SD, 6.0 years) and follow-up was ≈20 years. In contrast, our study sample was older (mean age, 68.4 years) and follow-up time shorter. Previous external validation studies of the CAIDE score have also found poor transportability when it is tested in older populations^14^ but not when looking at midlife risk for which it was developed.^12^ Second, it is possible that other variables that are not included in the CAIDE score such as those that are related to stroke or other vascular risk factors such as diabetes mellitus may also influence dementia risk.

With regard to the BDSI model, while the age distribution was similar between our study and the original development cohort, follow-up time differed (ie, 6 years in the development study versus ≈12–18 months in our study). Previous validation studies using general population-based samples have found comparable predictive accuracy during 2 years of follow-up.^13^ The BDSI includes age (1 point per year above age 65, up to 79 years of age), education (9 points), stroke (6 points), type 2 diabetes mellitus (3 points), body mass index (8 points), functional performance (ie, requiring assistance with money or medications, 10 points), and depressive symptoms or taking antidepressant medications (6 points). In the context of stroke, this points system may not accurately reflect the true impact of these risk variables, particularly with regard to diabetes mellitus, which has been shown to be associated with an increased risk of dementia^4,28^ but allocated a low number of points in the BDSI model. Future work could explore both recalibration of the model in stroke-specific populations and also to test the importance of other variables that might be independently associated with dementia,^28^ which could enhance predictive validity such as myocardial infarction and hypertension that are not included in this model.

The ANU-ADRI incorporates a wide range of variables, including age, sex, education, diabetes mellitus, smoking, and alcohol in the common variables model^14,19^ to predict Alzheimer disease. The extended model also includes other modifiable risk factors such as physical/cognitive activity and social network/engagement.^14^ The ANU-ADRI model’s predictive ability was low and similar to the BDSI but better than the CAIDE score, and we found in our stroke cohort that it showed similar discriminative accuracy to one of the development cohorts, although the C statistic representing the accuracy of prediction in that development cohort was also below the acceptable range. This may be because the ANU-ADRI model is not a midlife risk model and the mean age in the original testing cohorts at baseline tended to be older.^14^ However, this may also emphasize the importance of age as a risk factor, particularly given that previous studies did not find much difference between the risk prediction model and a model containing age alone.^13^

There has only been one model published for predicting poststroke dementia (Table VII in the Data Supplement).^15^ It includes age, occupation, number of strokes, left carotid vascular territory stroke location, admission NIH Stroke Scale score, admission Mini-Mental State Examination score, and admission Function Independence Measure motor score.^15^ The model correctly classified 93.4% of patients 3 months after stroke.^15^ We were not able to test this model due to a lack of key risk variables, including consistent magnetic resonance imaging findings, in our stroke cohort. Rather, for this study, we chose to externally validate general population-based dementia risk prediction models to determine whether a single dementia risk model could be applied. However, it is likely that acceptable predictive performance will require condition-specific data to be included. The identification of a single accurate model that had readily available variables would be of benefit to clinicians and researchers and could guide multidomain interventions.^29^ Our findings do not support the use of these general population-based dementia risk prediction models, however, and instead suggest that efforts should be made to develop stroke-specific models.

### Implications of Dementia Risk Prediction in Stroke

Overall, the results from our study suggest that dementia risk prediction scores developed in the general population do not work well in patients with stroke. This suggests that there are differences in risk factor profiles across disease groups. For instance, a stroke population is already at higher risk by virtue of the risk factors that led to the clinically evident cerebrovascular event. Some of these variables are already found in the dementia risk models we tested, so perhaps there is little remaining in these models to explain the additional heterogeneity in risk beyond the initial stroke event. Stroke-specific variables may be required for the development of new models in this population. They may include anatomic stroke location, type of stroke, and history of recurrent stroke, which have been found to be risk factors for poststroke dementia.^7^ Although neuroimaging variables have previously been found to add little to simple risk prediction models in dementia, this was at a population level.^18^ Neuroimaging variables such as white matter lesions, cerebral atrophy, and medial temporal lobe atrophy have been found to be risk factors for dementia after stroke and could be used along with stroke-specific variables in the development of stroke models.^30^ However, magnetic resonance imaging is not always possible and would have significant resource implications. Harmonization of stroke cohorts is both feasible and has the potential to be used in the development of future risk prediction models, but we need to ensure that there is uniformity in how we report individual variables.

Although many of the variables in existing models may already be optimized poststroke, earlier identification of those who may have a dementia illness ensures earlier access to appropriate care and provides information that some patients, families, and clinicians value for subsequent decisions. In the context of trials, it may be that specific (eg, multidomain) interventions could be used in those at higher risk of dementia to improve or even preserve cognitive function. This has certainly been the case in those risk stratified by the CAIDE model,^29^ and a similar approach could be trialed in stroke cohorts.

### Strengths and Limitations

Our study has a number of strengths. We used harmonized data from 4 stroke cohorts to increase sample size and, therefore, statistical power. Furthermore, given the range of available variables, we were able to test, for the first time, 3 different dementia risk prediction models in a stroke population. However, there are some limitations. First, some variables were modified because the specific data required for the risk models were not available (Table I in the Data Supplement). This could have reduced comparability between our study and the development studies. However, we did ensure that the substitutions were as close as possible to the original definitions. Second, although the predictive accuracy of the 3 models was clearly poor, the incident dementia rate across studies was generally low, leading to wide CIs. Third, the criteria for the diagnosis of dementia varied across cohorts, in particular, one cohort (CASPER) used VCD criteria to assess dementia and not a clinical rating. This could mean that individuals could be classed as minor VCD after they were classified as major VCD at subsequent follow-ups. Our analysis was similar to previous studies’ approach to the harmonization of dementia diagnoses across different cohorts (eg, see the COSMIC [Cohort Studies of Memory in an International Consortium] collaboration^31^). Fourth, the original models were generally developed in primarily white populations, which was also true in our study. We did include one South-East Asian population in the harmonized cohort for 2 of the models, although we did not specifically study the effects of ethnicity on the performance of those models. Finally, we undertook a complete case analysis, and this might have led to a select study population thereby affecting generalizability of the results. However, our sample size was large.

### Conclusions

The number of individuals at risk of poststroke dementia is increasing, but there is no validated condition-specific prediction tool. The dementia risk prediction models that we studied did not perform well in our stroke cohort, underlining the importance of developing a stroke-specific model, which will likely require the inclusion of a broader range of variables that are related to stroke and vascular risk factors and careful attention to their weightings. The next step is to use data from harmonized stroke cohorts to provide a large stroke-specific population for undertaking dementia risk prediction model development and external validation that is specific to this important subsample of individuals. We can then consider whether simple scores can be useful as the initial step in clinical monitoring of cognitive decline. The final model should permit the early identification of those individuals at the greatest risk of poststroke dementia and thus provide the opportunity for early targeted interventions.

## Acknowledgments

E.Y.H. Tang is funded by a National Institute for Health Research (NIHR) Doctoral Fellowship for this research project. This publication presents independent research funded by the NIHR. The views expressed are those of the author(s) and not necessarily those of the National Health Service (NHS), the NIHR, or the Department of Health and Social Care.

## Sources of Funding

E.Y.H. Tang is supported by a National Institute for Health Research (NIHR) Doctoral Research Fellowship (DRF-2015-08-006). Dr Robinson is supported by an NIHR Senior Investigator award (NF-SI-0616-10054). Dr Price receives support from a fellowship awarded by the Stroke Association, United Kingdom (TSA LECT 2017/03). The CU-STRIDE (Chinese University of Hong Kong–Stroke Registry Investigating Cognitive Decline) was supported by the Health and Health Services Research Fund (0708041) of the Food and Health Bureau of the Government of the Hong Kong Special Administrative Region. STROKOG (Stroke and Cognition Consortium) is funded by the Vincent Fairfax Family Foundation and the National Health and Medical Research Council of Australia Program Grant. Dr Köhler reports funding from the Adriana van Rinsum Ponsen Stichting (Dutch philantrophic fund).

## Disclosures

Dr Robinson reports grants from a National Institute for Health Research Senior Investigator award, outside the submitted work. Dr Sachdev reports a one-off payment from Biogen for an advisory board meeting and no conflicts in relation to this work. Dr Köhler reports a grants from the Adriana van Rinsum Ponsen Stichting (Dutch philantrophic fund). The other authors report no conflicts.

## Supplementary Material


