# Supplementary material for: Assessing the Predictive Validity of Simple Dementia Risk Models in Harmonized Stroke Cohorts
Source: Stroke. 2020 Jun 17;51(7):2095–102. doi: 10.1161/STROKEAHA.120.027473 (PMC7306263; doi:10.1161/STROKEAHA.120.027473)
Supplement: Supplementary file 1 [file str-51-2095-s001.pdf]

SUPPLEMENTAL MATERIAL

Online Supplementary Table I: Variable Substitution

Model	Original Model Variable	Score Assigned	Mapping to Harmonised Cohort
BDSI	Age	If 65 – 79 years, 1 point assigned per year above age 65	Those aged <65 were included, upper age limit was the same with those aged 79 excluded.
	Education of <12 years	9	CASPER cohort education variable was divided into low, middle and high categories. Educational level was scored in eight ordinal categories: (1) primary education, (2) lower vocational education, (3) intermediate general secondary education, (4) intermediate vocational education, (5) higher general secondary education, (6) higher vocational education, (7) higher professional education, and (8) university. Low = 1 and 2, Middle = 3 – 5, High = 6 – 8. >12 years education was equivalent to the high category.
	BMI <18.5 kg/m^2	8	As original
	Type 2 Diabetes	6	In EpiUSA, the type of diabetes was not specified. For the CASPER cohort, the type of diabetes was available but, due to a significant amount of missing data, both types of diabetes were included.
	History of Stroke	6	As original
	Needs help from others to manage money or medication	10	For the EpiUSA cohort, we used a variable representing only trouble handling money
	Currently takes antidepressant medications OR that "everything was an effort" ≥3 days per week over the past week	6	For EpiUSA, we relied on antidepressant use and for CASPER we relied on the Hospital Anxiety and Depression score (8 or over).
CAIDE	*Age:*<br><47 years<br>47-53 years<br>>53 years	 0<br>3<br>4	 As original
	Education:<br>≥10 years<br>7 – 9 years<br>0 – 6 years	 0<br>2<br>3	 As original
	Sex:<br>Women<br>Men	 0<br>1	 As original

	Systolic Blood Pressure:<br>≤140 mmHg<br>>140mmHg	 0<br>2	 As original
	Body Mass Index:<br>≤30 kg/m^2<br>>30 kg/m^2	 0<br>2	 As original
	Total Cholesterol:<br>≤6.5 mmol/L<br>>6.5 mmol/L	 0<br>2	We used the STROKDEM definition of hypercholesterolemia to represent high or low levels of cholesterol.
	Physical activity:<br>Active<br>Inactive	 0<br>1	We used each study's definition of whether the individual was physically active.
ANU-ADRI	*Age for males:*<br><65<br>65 – 69<br>70 – 74<br>75 – 79<br>80 – 84<br>85 – 89<br>≥90	 0<br>1<br>12<br>18<br>26<br>33<br>38	 As original
	Age for females:<br><65<br>65 – 69<br>70 – 74<br>75 – 79<br>80 – 84<br>85 – 89<br>≥90	 0<br>5<br>14<br>21<br>29<br>35<br>41	 As original
	Educational level (years):<br>< 8<br>8 – 11<br>>11	 0<br>3<br>6	In the CASPER dataset, we used their predefined low, middle and high categories to equate to the three categories used in the original model (see above).
	Diabetes:<br>No<br>Yes	 0<br>3	In the STRIDE cohort, this variable's coding was based on the use of a diabetes medication.
	Smoking:<br>Never smoked<br>Former smoker	 0<br>1	 As original

	Current smoker	4	
	Alcohol:<br>Abstainers<br>Light-to-moderate<br>Heavy	 0<br>-3<br>+3	For CASPER, <14 units per week was used as the cut off for light/moderate group. For STRIDE, we matched their "social drinker" category to the light-to-moderate category in the original model. The "regular drinker" category was categorised as the heavy group.<br>For Epi USA, we coded it as the following 1) One can of beer was equated to 12 ounces of 5% alcohol equivalent to 1.8 units 2) One glass of wine was equated to 5 ounces with 12 percent alcohol content equivalent to 1.8 units 3) One liquor is equated to 1.5 oz with 40% alcohol content equivalent to 1.8 units. Again <14 units per week was used as the cut off for the light/moderate group

Online Supplementary Table II: Contributions from Additional STROKOG Consortium Members

**STROKOG Members**
**We would like to thank the following for providing data but was not used in the final analysis due to missing key variables:**<br>*Bundang VCI Cohort (Korea):* Professor Hee-Joon Bae and Professor Jae-Sung Lim<br>*Cognitive Outcome After Stroke Cohort (Singapore):* Professor Christopher Chen and his team<br>*Cognitive Function After Stroke Cohort (UK):* Professor Raj Kalaria ad Professor Louise Allan<br>*Cracow Stroke Database (Poland):* Dr Aleksandra Klimkowicz-Mrowiec
**We would like to thank the following principal STROKOG Consortium members:**<br>Dr Rufus Akinyemi, Professor Philip Bath, Dr Amy Brodtmann, Dr Charlotte Cordonnier, Professor Martin Dichgans, Dr Abdel Douiri, Professor Olivier Godefroy, Dr Michael Hoffmann, Dr Hanna Jokinen, Dr Nagaendran Kandiah, Professor Frini Karayanidis, Dr Gary Lau, Professor Byung-Chul Lee, Dr Thomas Linden, Professor Hugh Stephen Markus, Professor Michael O'Sullivan, Dr Behnam Sabayan, Professor Velandai Srikanth, Professor Latchezar Traykov, Professor Joanna Wardlaw, Professor Qun Xu

Online Supplementary Table III. Demographic characteristics of those included compared to those excluded from the external validation analysis of each model

	**Included in the Analysis**	**Excluded from the Analysis**	**p-value**
**ANU-ADRI**			
N (%)	1065 (95.5)	50 (4.5)	-
N female (%)	463 (43.5)	25 (50)	0.363
N low educational attainment (%)	572 (53.7)	23 (46.0)	0.547
Mean age (SD)	68.6 (10.3)	71.2 (11.7)	0.084
**BDSI**			
N	282 (69.5)	124 (30.5)	-
N female (%)	106 (37.6)	63 (50.8)	**0.013**
N low educational attainment (%)	174 (61.7)	62 (50.0)	**0.028**
Mean age (SD)	65.9 (8.6)	67.4 (6.5)	0.093
**CAIDE**			
N	873 (80.7)	209 (19.3)	-
% female (n)	374 (42.8)	108 (51.7)	**0.021**

% low educational attainment (n)	391 (44.8)	88 (42.1)	0.672
Mean age (SD)	67.6 (10.9)	71.4 (9.1)	**<0.001**

Key Australian National University Alzheimer's Disease Risk Index (ANU-ADRI); BDSI Brief Dementia Screening Index; CAIDE Cardiovascular Risk Factors, Aging and Dementia (CAIDE) score ; SD Standard deviation

Bold text indicates where group differences were statistically significant i.e. p<0.05

Online Supplementary Table IV. BDSI - Harmonised Datasets for Total and First-Ever Stroke (EpiUSA and Casper)

	Total Sample			First-Ever Stroke Sample		
	**Total (n=406)**	**No Dementia (n=379) (n(%))**	**Incident Dementia (n=27) (n(%))**	**Total (n=288)**	**No Dementia (n=272) (n(%))**	**Incident Dementia (n=16) (n(%))**
**Age (mean (SD))**	66.4 (8.0)	66.0 (8.0)	71.2 (5.7)	65.9 (8.6)	65.6 (8.7)	69.9 (5.9)
**Education (n(%))**						
- **High Education or >=12 years**	170 (41.9)	158 (41.7)	12 (44.4)	111 (38.5)	105 (38.6)	6 (37.5)
- **Low/Middle Education or < 12 years**	236 (58.1)	221 (58.3)	15 (55.6)	177 (61.5)	167 (61.4)	10 (62.5)
**BMI (n(%))**						
- **≥18.5**	316 (77.8)	300 (79.2)	16 (59.3)	228 (79.2)	219 (80.5)	9 (56.3)
- **<18.5**	6 (1.5)	4 (1.1)	2 (7.4)	3 (1.0)	2 (0.7)	1 (6.3)
- **Missing variable**	84 (20.7)	75 (19.8)	9 (33.3)	57 (19.8)	51 (18.8)	6 (37.5)
**Stroke (n(%))**	406 (100)	379 (100)	27 (100)	288 (100)	272 (100)	16 (100)
**Diabetes (n(%))**						
- No	306 (75.4)	291 (76.8)	15 (55.6)	229 (79.5)	221 (81.3)	8 (50.0)
- Yes	100 (24.6)	88 (23.2)	12 (44.4)	59 (20.5)	51 (18.8)	8 (50.0)
**Help Required for Money or Medications (n(%))**						
- No	306 (75.4)	292 (77.0)	14 (51.9)	217 (75.4)	210 (77.2)	7 (43.8)
- Yes	86 (21.2)	73 (19.3)	13 (48.1)	60 (20.8)	51 (18.8)	9 (56.3)
- Missing variable	14 (3.5)	14 (3.7)	0 (0.0)	11 (3.8)	11 (4.0)	0 (0.0)
**Depression (n(%))**						
- No	292 (71.9)	276 (72.8)	16 (59.3)	205 (71.1)	197 (72.4)	8 (50.0)
- Yes	40 (9.9)	38 (10.0)	2 (7.4)	36 (12.5)	34 (12.5)	2 (12.5)
- Missing variable	74 (18.2)	65 (17.2)	9 (33.3)	47 (16.3)	41 (15.1)	6 (37.5)
**Complete Cases (n (%))**	282 (69.5)	266 (70.2)	16 (59.3)	207 (71.9)	197 (72.4)	10 (62.5)
**Missing Scores (n (%))**	124 (30.5)	113 (29.8)	11 (40.7)	81 (28.1)	75 (27.6)	6 (37.5)
**Follow-up Time in Days (Complete Cases) (Mean (SD), Range)**	336.0 (51.7), (176 – 490)	338.3 (49.7), (192 – 490)	298.7 (69.7), (176 – 434)	345.6 (46.8) (192 – 490)	347.9 (45.2) (192 – 490)	299.2 (55.8) (203 – 376)

SD, standard deviation

Online Supplementary Table V. ANU-ADRI (Common Variables Model) – Harmonised Datasets for Total and First-Ever Stroke (Epi USA, Casper and STRIDE)

	Total Sample			First-Ever Stroke Sample		
	**Total (n=1115)**	**No Dementia (n=1059) (n(%))**	**Incident Dementia (n=56) (n(%))**	**Total (n=864)**	**No Dementia (n=828) (n(%))**	**Incident Dementia (n=36) (n(%))**
**Age (mean (SD))**	68.7 (0.3)	68.3 (10.3)	75.9 (8.9)	68.1 (10.7)	67.8 (10.6)	74.7 (9.5)
**Sex (n (%))**						
- Female	488 (43.8)	452 (42.7)	36 (64.3)	372 (43.1)	353 (42.6)	19 (52.8)
- Male	627 (56.2)	607 (57.3)	20 (35.7)	492 (56.9)	475 (57.4)	17 (47.2)
**Education (n (%))**						
- Low/Less than 8 years	595 (53.4)	561 (53.0)	34 (60.7)	466 (53.9)	444 (53.6)	22 (61.1)
- Middle/8-11 years	258 (23.1)	250 (23.6)	8 (14.3)	210 (24.3)	204 (24.6)	6 (16.7)
- High/Greater than 11 years	262 (23.5)	248 (23.4)	14 (25.0)	188 (21.8)	180 (21.7)	8 (22.2)
**Alcohol**						
- None	777 (69.7)	729 (68.9)	2 (3.6)	603 (69.8)	570 (68.8)	33 (91.7)
- Light-Moderate	184 (16.5)	182 (17.2)	48 (85.7)	171 (19.8)	171 (20.7)	0 (0.0)
- Heavy Drinker	105 (9.4)	102 (9.6)	3 (5.4)	85 (9.8)	82 (9.9)	3 (8.3)
- Missing variable	49 (4.4)	46 (4.3)	3 (5.4)	5 (0.6)	5 (0.6)	0 (0.0)
**Diabetes (n (%))**						
- No	770 (69.1)	736 (69.5)	34 (60.7)	612 (70.8)	592 (71.5)	20 (55.6)
- Yes	345 (30.9)	323 (30.5)	22 (39.3)	252 (29.2)	236 (28.5)	16 (44.4)
**Smoking (n (%))**						
- Never Smoked	547 (49.1)	517 (48.8)	30 (53.6)	435 (50.4)	415 (50.1)	20 (55.6)
- Former Smoker	407 (36.5)	389 (36.7)	18 (32.1)	298 (34.5)	289 (34.9)	9 (25.0)
- Current Smoker	157 (14.1)	149 (14.1)	8 (14.3)	128 (14.8)	121 (14.6)	7 (19.4)
- Missing variable	4 (0.4)	4 (0.4)	0 (0)	3 (0.4)	3 (0.4)	0 (0.0)
**Complete Cases (n (%))**	1065 (95.5)	1012 (95.6)	53 (94.6)	858 (99.3)	822 (99.3)	36 (100.0)
**Missing Scores (n (%))**	50 (4.5)	47 (4.4)	3 (5.4)	6 (0.7)	6 (0.7)	0 (0.0)
**Follow-up Time in Days (Complete Cases) (Mean (SD), Range)**	362.4 (54.2), (134 – 560)	363.6 (52.4), (134 – 560)	340.8 (78.4), (176 – 531)	366.0 (49.6), (134 – 541)	367.2 (47.7), (134 – 541)	337.1 (76.4), (198 – 531)

SD, standard deviation

Online Supplementary Table VI. CAIDE 1 Model – Harmonised Datasets for Total and First-Ever Stroke (Epi USA, STRIDE and STROKDEM)

	Total Sample			First-Ever Stroke Sample		
	**Total (n=1082)**	**No Dementia (n=1019)**	**Incident Dementia (n=63)**	**Total (n=843)**	**No Dementia (n=800)**	**Incident Dementia (n=43)**
**Age (mean (SD))**	68.4 (10.7)	67.9 (10.6)	75.3 (9.3)	67.7 (11.0)	67.3 (10.9)	74 .2 (10.1)
**Sex (n (%))**						
- Female	482 (44.6)	442 (43.4)	40 (63.5)	367 (43.5)	344 (43.0)	23 (53.5)
- Male	600 (55.5)	577 (56.6)	23 (36.5)	476 (56.5)	456 (57.0)	20 (46.5)
**Education (n (%))**						
- ≥10 years	396 (36.6)	375 (36.8)	21 (33.3)	306 (36.3)	292 (36.5)	14 (32.6)
- 7 – 9 years	207 (55.7)	196 (19.2)	11 (17.5)	161 (19.1)	152 (19.0)	9 (20.9)
- 0 – 6 years	479 (44.3)	448 ()	31 (49.2)	376 (44.6)	356 (44.5)	20 (46.5)
**Hypercholesterolaemia/Total Cholesterol >6.5mmol**						
- No	786 (72.6)	746 (73.2)	40 (63.5)	632 (75.0)	605 (75.6)	27 (62.8)
- Yes	187 (17.3)	175 (17.2)	12 (19.0)	142 (16.8)	132 (16.5)	10 (23.3)
- Missing variable	109 (10.1)	98 (9.6)	11 (17.5)	69 (8.2)	63 (7.9)	6 (14.0)
**Systolic Blood Pressure >140mmHg**						
- No	354 (32.7)	340 (33.3)	14 (22.2)	282 (33.5)	274 (34.3)	8 (18.6)
- Yes	622 (57.5)	585 (57.4)	37 (58.7)	489 (58.0)	460 (57.5)	29 (67.4)
- Missing variable	106 (9.8)	94 (9.2)	12 (19.0)	72 (8.5)	66 (8.3)	6 (14.0)
**Physically Active (n (%))**						
- Yes	715 (66.1)	676 (66.3)	39 (61.9)	544 (64.5)	520 (65.0)	24 (55.8)
- No	310 (28.7)	293 (28.8)	17 (27.0)	260 (30.8)	247 (30.9)	13 (30.2)
- Missing variable	57 (5.3)	50 (4.9)	7 (11.1)	39 (4.6)	33 (4.1)	6 (14.0)
**BMI >30 (n (%))**						
- No	795 (73.5)	758 (74.4)	37 (58.7)	634 (75.2)	608 (76.0)	26 (60.5)
- Yes	103 (9.5)	96 (9.4)	7 (11.1)	82 (9.7)	76 (9.5)	6 (14.0)
- Missing variable	184 (17.0)	165 (16.2)	19 (30.2)	127 (15.1)	116 (14.5)	11 (25.6)
**Complete Cases (n (%))**	873 (80.7)	830 (81.5)	43 (68.3)	700 (83.0)	669 (83.6)	31 (72.1)
**Missing Scores (n (%))**	209 (19.3)	189 (18.5)	20 (31.7)	143 (17.0)	131 (16.4)	12 (27.9)
**Follow-up Time in Days (Complete Cases) (Mean (SD), Range)**	370.4 (51.0), (176 – 645)	370.9 (49.0), (192 – 645)	359.3 (80.3), (176 – 593)	373.7 (45.3), (192 – 645)	374.5 (43.9), (192 – 645)	357.2 (67.3), (203-531)

Key SD standard deviation

Online Supplementary Table VII: Post-Stroke Models for Cognitive Impairment and Dementia

Authors (number of participants in development study)	Variables	Model Discrimination Performance	Follow-up Time	Outcome
J-H Lin et al(1) (n=283)	Age, occupation, number of strokes, left carotid vascular territory stroke location, admission NIH Stroke Scale score, admission Mini-Mental State Examination score, admission Function Independence Measure motor score	Correct classification of 93.4% of patients	3 months	Dementia
Kandiah et al(2) (n=209)	Age, education, acute cortical infarcts, white matter hyperintensity, chronic lacunes, global cortical atrophy and intracranial large vessel stenosis	AUC = 0.83 (95%CI: 0.77 – 0.88)	3-6 months	Cognitive Impairment
Chander et al(3) (n=209)	Chronic lacunes, hyperintensities in white matter regions, age, non-lacunar cortical infarct (acute), global cortical atrophy, education	AUC = 0.82 (95%CI: 0.76 – 0.88)	3-6 months	Cognitive Impairment

Key

95%CI 95 percent confidence interval; **AUC** Area under the Curve

References

1. Lin JH, Lin RT, Tai CT, Hsieh CL, Hsiao SF, Liu CK. Prediction of poststroke dementia. Neurology. 2003;61(3):343-8.
2. Kandiah N, Chander RJ, Lin X, Ng A, Poh YY, Cheong CY, et al. Cognitive Impairment after Mild Stroke: Development and Validation of the SIGNAL2 Risk Score. Journal of Alzheimer's disease : JAD. 2016;49(4):1169-77.
3. Chander RJ, Lam BYK, Lin X, Ng AYT, Wong APL, Mok VCT, et al. Development and validation of a risk score (CHANGE) for cognitive impairment after ischemic stroke. Scientific reports. 2017;7(1):12441.
